# Extracellular Recognition of Oomycetes during Biotrophic Infection of Plants

**DOI:** 10.3389/fpls.2016.00906

**Published:** 2016-06-21

**Authors:** Tom M. Raaymakers, Guido Van den Ackerveken

**Affiliations:** Plant–Microbe Interactions, Department of Biology, Faculty of Science, Utrecht University, UtrechtNetherlands

**Keywords:** oomycete pathogens, pattern recognition, MAMP/DAMP, plant disease resistance, secreted proteins, extracellular recognition

## Abstract

Extracellular recognition of pathogens by plants constitutes an important early detection system in plant immunity. Microbe-derived molecules, also named patterns, can be recognized by pattern recognition receptors (PRRs) on the host cell membrane that trigger plant immune responses. Most knowledge on extracellular pathogen detection by plants comes from research on bacterial and fungal pathogens. For oomycetes, that comprise some of the most destructive plant pathogens, mechanisms of extracellular pattern recognition have only emerged recently. These include newly recognized patterns, e.g., cellulose-binding elicitor lectin, necrosis and ethylene-inducing peptide 1-like proteins (NLPs), and glycoside hydrolase 12, as well as their receptors, e.g., the putative elicitin PRR elicitin response and the NLP PRR receptor-like protein 23. Immunity can also be triggered by the release of endogenous host-derived patterns, as a result of oomycete enzymes or damage. In this review we will describe the types of patterns, both pathogen-derived exogenous and plant-derived endogenous ones, and what is known about their extracellular detection during (hemi-)biotrophic oomycete infection of plants.

## Introduction

Most plant pathogens are able to penetrate host tissues but essentially grow in the plant apoplast or extracellular space. Even haustoria, feeding structures formed by many biotrophic fungi and oomycetes that invaginate host cells, remain separated from the plant cell cytoplasm by the plant-derived extrahaustorial membrane (Parniske, 2000). It, therefore, comes as no surprise that a first line of pathogen recognition is extracellular and mediated by membrane-bound receptors that detect microbe- or host damage-derived molecules or patterns. Over the last decades, many pattern-recognition receptors mediating immunity to patterns of bacteria and fungi have been reported. Well known examples include the *Arabidopsis* receptor-like kinase (RLK) FLAGELLIN-SENSITIVE 2 (FLS2) that mediates recognition of bacterial flagellin, and the RLK, CHITIN ELICITOR RECEPTOR KINASE 1 (CERK1) involved in detection of fungal chitin (Zipfel, 2014). Flagellin and chitin are considered microbe-associated molecular patterns (MAMPs), while their cognate receptors are termed pattern-recognition receptors (PRRs; Jones and Dangl, 2006; Hein et al., 2009; Dodds and Rathjen, 2010). MAMPs are generally considered conserved molecules that occur in all species of a given taxon. There are, however, many examples of patterns that are species-specific or that are less well conserved, e.g., apoplastic effectors that are recognized by cognate resistance gene-encoded membrane-bound receptors (Thomma et al., 2011). In this review we, therefore, refer to all extracellular molecules that trigger immunity as patterns (Cook et al., 2015). In older papers the term “elicitor” is most often used, but many of these can be regarded as patterns too (Boller and Felix, 2009; Cook et al., 2015). Although numerous oomycete patterns have been described, knowledge on the mechanism of their extracellular recognition has only emerged recently for some of them.

Oomycetes are filamentous organisms that belong to the Stramenopiles, a taxon that also encompasses the diatoms and brown algae. Many oomycetes are free-living saprobes in soils or aquatic environments. The best known oomycetes, or the most infamous ones, are species that are pathogenic on plants, e.g., the potato late blight pathogen *Phytophthora infestans* and the grape downy mildew *Plasmopara viticola* (Haas et al., 2009; Kamoun et al., 2015). Five main taxa of phytopathogenic oomycetes can be distinguished: (i) the genus *Phytophthora*, (ii) the downy mildews, (iii) the white blister rusts, (iv) the genus *Pythium*, and (v) the genus *Aphanomyces* (Thines and Kamoun, 2010).

**FIGURE 1:**
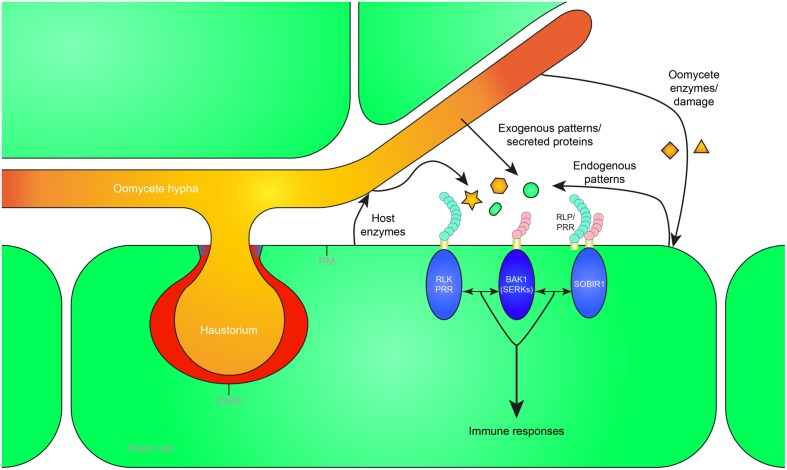
**Recognition of exogenous and endogenous patterns during oomycete infection leads to the activation of plant immunity.** Oomycete pathogens secrete proteins in the apoplast (white) and extrahaustorial matrix (red) which can be perceived as exogenous patterns by pattern recognition receptors (PRRs) in the plant plasma membrane (PM) or extrahaustorial membrane (EHM). Furthermore, pathogen-derived cell wall or membrane fragments are released during infection, possibly by host enzymes, and recognized as patterns by the host. Mechanical damage or damage caused by oomycete secreted enzymes can release endogenous patterns that trigger immunity. The receptor-like kinase (RLK) BRI1-ASSOCIATED RECEPTOR KINASE 1 (BAK1), a member of the SOMATIC EMBRYOGENESIS RECEPTOR KINASE (SERK) family, functions as a central hub of RLK and receptor-like protein (RLP) triggered immunity. RLPs form a bimolecular receptor kinase with the RLK SUPPRESSOR OF BIR1 1 (SOBIR1). RLKs and RLPs bound to SOBIR1 associate with BAK1 to activate pattern-triggered immunity upon perception of exogenous or endogenous patterns. The haustorial callosic neckband that is sometimes formed in oomycete–plant interactions is depicted in blue. Oomycete-derived patterns and proteins are depicted in orange, plant-derived patterns in green.

In this review, we focus on the extracellular recognition of (hemi-)biotrophic oomycetes, on patterns that trigger immunity, and on mechanisms of pattern recognition. A broad range of molecules or patterns are released during oomycete infection of plants, either exogenous ones derived from the pathogen, or endogenous ones that are released from the plant host (**Figure 1**). The distinction between exogenous and endogenous signals can also be referred to as non-self and modified-self patterns (Schwessinger and Zipfel, 2008). Endogenous patterns, also known as damage-associated molecular patterns (DAMPs), either result from oomycete enzyme activities, or from lysis or disruption of host cells during the infection process. Oomycete patterns and other elicitors can be grouped based on their cellular origin (oomycete cell wall/membrane, or pathogen secreted). We will review the different patterns, their cellular origin, and what is known about the detection mechanisms that have evolved to recognize such patterns, and trigger the plant immune system.

## Oomycete Patterns Triggering Immunity

Plants can sense a wide variety of extracellular oomycete-derived patterns. These molecules can be secreted by oomycetes during infection, or released from the invading pathogens by host-derived enzymes (**Table 1**). Several oomycete patterns are derived from the pathogen’s cell wall or membrane, whereas others are secreted to the extracellular environment before being detected by the plant immune system. Below we discuss the different extracellular patterns, where they derive from, and what is known about their function.

**Table 1:** Oomycete patterns that activate plant immunity.

Elicitor^a^	Source	Type	(Putative) Receptor^b^	Receptor type^c^	Co-receptors^d^	Reference
β-Glucans	Cell wall	Carbohydrate	GBP, additional components required	GH16		Fesel and Zuccaro, 2015
Glucan-chitosaccharides	Cell wall	Carbohydrate	Unknown			Nars et al., 2013
Pep-13	Cell wall	Peptide	Unknown monomeric 100 kDa integral plasma membrane protein			Reiss et al., 2011
Eicosapolyenoic acids	Membrane	Fatty acid	Unknown			Robinson and Bostock, 2015
GH12 (XEG1)	Secreted protein	Protein	Unknown		SERK3/BAK1 required	Ma et al., 2015
nlp20/nlp24	Secreted protein	Peptide	RLP23	RLP	BAK1 and SOBIR1 required	Albert et al., 2015
Elicitins	Secreted protein	Protein	ELR	RLP	BAK1 and SOBIR1 required	Du et al., 2015
CBM1/CBEL	Secreted protein	Protein	Unknown		partially requires BAK1	Larroque et al., 2013
OPEL	Secreted protein	Protein	Unknown			Chang et al., 2015

*^a^GH12 = glycoside hydrolase family 12; XEG1 = xyloglucanspecific endo-β-1,4-glucanase; nlp = necrosis and ethylene-inducing peptide 1-like protein; CBM1 = carbohydrate binding module 1; CBEL = cellulose-binding elicitor lectin.*

*^b^GBP = Glucan Binding Protein; RLP23 = receptor-like protein 23; ELR = elicitin response.*

*^c^GH16 = glycoside hydrolase family 16; RLP = receptor-like protein.*

*^d^SERK3 = SOMATIC EMBRYOGENESIS RECEPTOR-LIKE KINASE 3; BAK1 = BRI1-ASSOCIATED RECEPTOR KINASE; SOBIR1 = SUPPRESSOR OF BIR1 1.*

### Cell Wall/Membrane-Derived Patterns

#### β-Glucans

The most abundant constituents of oomycete cell walls are glucans, polysaccharides that consist of linked glucose units (Aronson et al., 1967; Sietsma et al., 1969). β-1,3 and β-1,6-glucan are the major components of oomycete cell walls, whereas cellulose, a β-1,4-glucan, forms a relatively small fraction (Aronson et al., 1967). β-1,6-Glucan is only found in oomycetes and fungi, whereas cellulose and β-1,3-glucan are present in plant cell walls too (Fesel and Zuccaro, 2015).

A β-glucan-triggered response, i.e., the accumulation of the phytoalexin glyceollin, was first observed when soybean (*Glycine max*) was treated with glucans isolated from cell walls of *Phytophthora sojae* (previously *P. megasperma* f. sp. *glycinea* and *P. megasperma* var. *sojae*) (Ayers et al., 1976). β-Glucans also trigger phytoalexin production in several other fabaceous species, and in potato (*Solanum tuberosum*), although this is a weaker response (Cline et al., 1978; Cosio et al., 1996). A purified β-1,3/1,6-glucan heptaglucoside was found to be one of the active molecules in eliciting production of phytoalexins in soybean (Sharp et al., 1984a,b). Laminarin, an oligomeric β-1,3-glucan with β-1,6-glucan branches isolated from the marine brown alga *Laminaria digitata*, is another pattern that can induce a plethora of defense-associated responses in *Nicotiana tabacum*, grapevine (*Vitis vinifera*), and the monocots rice (*Oryza sativa*) and wheat (*Triticum aestivum*; Inui et al., 1997; Klarzynski et al., 2000; Aziz et al., 2003). Furthermore, *Arabidopsis thaliana* is responsive to the β-glucan laminarin, although it does not respond to the oomycete-derived heptaglucoside elicitor. *Arabidopsis* responses to laminarin are mediated by the plant hormone ethylene, and do not seem to involve the well-known defense hormone salicylic acid (SA). In contrast, when *Arabidopsis* or tobacco plants are treated with a sulfated form of laminarin the expression of the SA-induced marker gene *PR1* is induced (Ménard et al., 2004). Taken together, responses to β-glucans vary greatly depending on the specific β-glucan and plant species. Therefore, different plant species might have different receptors involved in the recognition of different β-glucan patterns.

*Phytophthora*-derived β-1,3-glucan was shown to bind soybean membranes (Yoshikawa et al., 1983). The glucan-binding protein (GBP) from soybean was identified and it was demonstrated that, when expressed in tobacco and *Escherichia coli*, GBP conferred β-glucan-binding activity. Furthermore, an antibody raised against GBP inhibited β-glucan-binding activity in soybean and reduced phytoalexin accumulation (Umemoto et al., 1997). Interestingly, GBP also shows β-glucanase activity and might release β-glucans from the pathogen’s cell wall (Fliegmann et al., 2004). After heterologous expression of soybean GBP in tomato, high affinity binding of the β-1,3/1,6-glucan heptaglucoside was observed. However, this did not result in activation of downstream defense responses in tomato (Mithöfer et al., 2000; Fliegmann et al., 2004). These data suggest that additional, probably membrane-bound, proteins are required to recognize the β-glucan patterns (Mithöfer et al., 2000).

#### Glucan-Chitosaccharides

Recently, glucan-chitosaccharides were isolated from the cell wall of the root oomycete *Aphanomyces euteiches* and were found as novel patterns that triggered calcium oscillations in the nucleus of root cells and induced defense genes in *Medicago truncatula* (Nars et al., 2013). How these molecules are perceived is not yet known, but there is a role for the nod factor perception (NFP) protein, a lysin motif (LysM)-RLK. NFP is involved in the recognition of microbial *N*-acetylglucosamine patterns and is required for nodule formation in interaction with *Rhizobium* bacteria. An *nfp* mutant was more susceptible to *A. euteiches*, whereas overexpression of *NFP* led to increased resistance, demonstrating its involvement in the perception of *A. euteiches* by *M. truncatula* (Rey et al., 2013). However, NFP was not required for the glucan-chitosaccharide-induced calcium oscillations, suggesting a regulatory function in defense for NFP rather than direct recognition (Nars et al., 2013).

#### Transglutaminases (Pep-13)

Transglutaminases (TGases) are a widespread family of enzymes, found in prokaryotes and eukaryotes, that facilitate cross-linking between glutamine and lysine residues in proteins, thereby strengthening structures, e.g., cell walls (Lorand and Graham, 2003; Martins et al., 2014). The formation of a covalent bond between amino acid residues confers high resistance to proteolysis (Reiss et al., 2011). In oomycetes, TGases could protect cell walls from hydrolytic host enzymes. A 42-kDa TGase cell wall glycoprotein (GP42) of *P. sojae* functions as a potent elicitor of phytoalexin synthesis in the non-host parsley (*Petroselinum crispum*) (Parker et al., 1991). A 13-amino acid peptide fragment (Pep-13) derived from GP42 was found responsible for triggering immunity and was shown to bind to purified plasma membranes of parsley. Furthermore, Pep-13 elicits a multitude of defense responses, e.g., expression of defense-related genes and phytoalexin production (Nürnberger et al., 1994, 1995; Hahlbrock et al., 1995). Interestingly, Pep-13 treatment of potato resulted in a similar defense activation, with the distinct difference that it induced a hypersensitive response (HR; Halim et al., 2004).

GP42 homologs are only found in oomycetes and some marine bacteria belonging to the genus *Vibrio* that are pathogenic on fish and several marine invertebrates (Reiss et al., 2011). It is thought that an ancestral oomycete, from which species of *Phytophthora*, *Pythium* and downy mildews have evolved, acquired GP42 from *Vibrio* bacteria through horizontal gene transfer, giving an selective advantage over oomycetes that lack this TGase (Reiss et al., 2011). A 100 kDa monomeric plasma membrane protein from parsley was shown to bind to the Pep-13 ligand and thus may be part of the putative receptor complex (Nennstiel et al., 1998).

#### Eicosapolyenoic Acids

Application of mycelial extracts from *P. infestans* to potato tubers led to necrosis and accumulation of phytoalexins, predominantly rishitin, and lubimin. The molecules responsible for triggering this response were identified as the eicosapolyenoic acids (EPs), arachidonic acid (AA), and eicosapentaenoic acid (EPA; Bostock et al., 1981). Treating potato tuber slices with AA greatly reduced or even arrested growth of *P. infestans* (Bostock et al., 1982). EPs are components of *Phytophthora* cells and are seemingly not present in other microbial classes nor are they produced by higher plants (Robinson and Bostock, 2015). Interestingly, the downy mildew *Hyaloperonospora arabidopsidis* has lost the genes required for AA synthesis (Baxter et al., 2010). It is tempting to speculate that *H. arabidopsidis* has lost this ability through evolution as a way to avoid recognition.

Eicosapolyenoic acids induce the accumulation of antimicrobial compounds in many plant species, ranging from many solanaceous species, e.g., potato and tomato, to bean (*Phaseolus vulgaris*) and avocado (*Persea americana*) (Longland et al., 1987; Romero-Correa et al., 2014; Robinson and Bostock, 2015). Furthermore, in potato application of AA induced accumulation of reactive oxygen species (ROS), that could be involved in mediating synthesis of the phytoalexin rishitin from lubimin (Yoshioka et al., 2001).

EPs are able to trigger systemic acquired resistance in several plants species to different pathogens. The hormonal regulation of these responses seems to differ among plant species; in some the SA pathway is elicited, whereas in other species responses seem to rely on jasmonic acid (JA) or ethylene. It is postulated that this may be due to the concentration of EPs in the treatment (Robinson and Bostock, 2015). For example, *Arabidopsis* plants made to produce low levels of EP showed increased resistance to *Botrytis cinerea*, *P. capsici* and aphid feeding, but higher susceptibility to *Pseudomonas syringae* pv. *tomato* (DC3000). This was associated with higher levels of JA and enhanced expression of JA-related genes, but decreased SA levels and reduced expression of SA-related genes. Furthermore, low levels of AA administered to tomato leaves resulted in increased JA levels and decreased SA levels and higher resistance against *Botrytis cinerea* (Savchenko et al., 2010).

How, exactly, EPs are perceived remains to be resolved. EPs could be recognized directly by a membrane-bound receptor, leading to the activation of plant immunity. Another possibility is that plant membranes that readily incorporate AA (Ricker and Bostock, 1992), are perturbed leading to the release of endogenous patterns from the host cell cytoplasm. Or alternatively, AA can be used as a substrate for lipoxygenases, e.g., the potato LOX1, thereby producing oxylipin signals that trigger plant immunity. In the latter two scenarios recognition would be independent of plant PRRs (Robinson and Bostock, 2015).

Interestingly, treating potato with a combination of AA and β-1,3-glucans strongly increased the response to AA. β-glucans alone, however, did not trigger a response in potato (Preisig and Kuć, 1988).

### Secreted Proteins

#### Glycoside Hydrolase 12 Proteins

Recently, the XEG1 (xyloglucanspecific endo-β-1,4-glucanase) protein was isolated from *P. sojae* culture filtrates (Ma et al., 2015). This secreted protein elicits cell death in *N. benthamiana*, *N. tabacum*, pepper (*Capsicum annuum*), tomato (*S. lycopersicon*) and soybean but not in maize (*Zea mays*) and cotton (*Gossypium hirsutum*). Analysis of the XEG1 protein sequence revealed that it belongs to the glycoside hydrolase GH12 family that is widespread amongst prokaryotic and eukaryotic microbes, especially in plant-associated microorganisms. Within the *Phytophthora* genus many GH12 proteins are found of which half trigger cell death in *N. benthamiana.* The downy mildew *H. arabidopsidis* also has three GH12 genes, however, none of them encode a protein that elicits cell death (Ma et al., 2015). Previously, it was demonstrated that fungal GH12 proteins are able to degrade β-glucan (Karlsson et al., 2002) and xyloglucan, a hemicellulose found in the plant cell wall (Master et al., 2008). Recombinant XEG1 protein partially released reducing sugars from both glucans, but was most active with a xyloglucan substrate. Mutations in the catalytic site of XEG1 strongly decreased xyloglucanase activity and abolished β-glucanase activity. In contrast, XEG1 enzyme activity was not required for the induction of cell death in *N. benthamiana* and soybean. Moreover, active and inactive recombinant XEG1 were able to induce resistance against *P. sojae* and *Phytophthora parasitica* var. *nicotianae* to a similar extent in soybean and *N. benthamiana*, respectively. Silencing as well as overexpression of XEG1 in *P. sojae* both led to reduced virulence on soybean through distinct mechanisms. Silenced *P. sojae* lines showed reduced virulence, but did not activate a stronger defense response in soybean, suggesting that XEG1 has a role in virulence, possibly through breakdown of cell wall components. XEG1 overexpression transformants induced more ROS accumulation and callose deposition compared to wildtype *P. sojae*, confirming the idea that XEG1 acts as a MAMP. A XEG1 PRR has not been identified but XEG1 requires the co-receptor SOMATIC EMBRYOGENESIS RECEPTOR-LIKE KINASE 3/BRI1-ASSOCIATED RECEPTOR KINASE (SERK3/BAK1) for triggering cell death, suggesting that a SERK3/BAK1-associated RLK or RLP recognizes XEG1 (Ma et al., 2015).

#### Necrosis and Ethylene-Inducing Peptide 1 (Nep1)-Like Proteins

Necrosis and ethylene-inducing peptide 1 (Nep1)-like proteins (NLPs) form a family of secreted proteins mainly found in plant-associated microorganisms, and cytotoxic members are well known to induce necrosis and ethylene production in dicot plants (Bailey, 1995; Oome and Van den Ackerveken, 2014). Three types of NLPs have been identified: type 1 NLPs are found in bacteria, oomycetes and fungi, type 2 NLPs are found in fungi and bacteria and the newly identified type 3 NLPs are only present in fungi (Oome and Van den Ackerveken, 2014). Although many members of the NLP family are cytotoxic to plants, in recent years many non-cytoxic NLPs have been identified in fungal and oomycete species with a (hemi-)biotrophic lifestyle (Cabral et al., 2012; Dong et al., 2012; Zhou et al., 2012). In search of the function of 10 non-cytotoxic NLPs of the obligate biotrophic downy mildew *H. arabidopsidis* (HaNLPs) it was found that NLPs activate plant immunity in *Arabidopsis* (Oome et al., 2014). Expression of *HaNLPs* in *Arabidopsis* led to a severe growth reduction and increased resistance to *H. arabidopsidis* for 7 out of 10 HaNLPs. Only a small fragment of the tested HaNLP3 protein was sufficient to activate plant defense responses and immunity to downy mildew. This 20–24 amino acid fragment (nlp20/nlp24) contains two conserved regions. The second region is the heptapeptide motif GHRHDWE which is highly conserved in all NLPs (Oome and Van den Ackerveken, 2014). The first motif that starts with the AIMY amino acid sequence is highly conserved in type 1 NLPs (Oome et al., 2014). Treatment of *Arabidopsis* plants with synthetic nlp24 peptides corresponding to an oomycete, fungal and bacterial type 1 NLP resulted in the increased production of the defense-related phytohormone ethylene and high resistance to downy mildew. Conversely, a synthetic peptide of a type 2 NLP from the bacterial pathogen *Pectobacterium carotovorum* that lacks the AIMY motif was unable to elicit a response in *Arabidopsis*. Taken together, this demonstrated that the first motif contains the immunogenic part of nlp24 (Oome et al., 2014). Furthermore, nlp20, a peptide based on *Pp*NLP, a cytotoxic *P. parasitica* type 1 NLP, was sufficient for MAPK activation, production of ROS, and increased callose deposition in *Arabidopsis*, but did not have any cytotoxic effect (Böhm et al., 2014). Other plant species were tested for their ability to respond to nlp peptides, revealing that nlp-triggered ethylene production was observed in several closely related Brassicaceae species, and also in more distantly related lettuce plants (*Lactuca sativa*), but not in solanaceous species such as tomato, potato, and *N. benthamiana* (Böhm et al., 2014).

In a screen for nlp20 sensitivity, a collection of T-DNA insertion mutants corresponding to 29 RLKs and 44 RLPs were tested for loss of nlp20-induced ethylene production. Furthermore, 135 natural accessions of *Arabidopsis* were also tested for the loss of nlp20 sensitivity. Two T-DNA insertion alleles of *RLP23*, *rlp23-1*, and *rlp23-2* that were unable to express the receptor-like protein as well as three *Arabidopsis* accessions that carried a frameshift mutation resulting in a premature stop codon in *RLP23* coding sequence were insensitive to nlp20. It was shown that the RLP23 LRR domain physically interacts with nlp20 *in vitro* and *in planta* (Albert et al., 2015). RLP23 lacks a cytoplasmic signaling domain but was shown to require the RLK SUPPRESSOR OF BIR1 1 (SOBIR1) for signaling. RLP23 and SOBIR1 interact in the absence of nlp peptides (Bi et al., 2014; Albert et al., 2015), whereas a second RLK, BAK1, was recruited only in presence of the ligand (Albert et al., 2015). *Arabidopsis sobir1* and *bak1-5/bkk1* mutants lost nlp20-responsiveness, indicating that SOBIR1 and BAK1 are required for RLP23 to function. Moreover, it was demonstrated that RLP23 is required for nlp peptide-induced resistance. Unlike wildtype *Arabidopsis*, nlp24-treatment of *rlp23* mutants did not result in an increased resistance to *H. arabidopsidis* (Albert et al., 2015).

#### Elicitins

Many oomycete pathogens secrete small 10 kDa proteins called elicitins. The first proteins from this family identified were cryptogein and capsicein from *Phytophthora cryptogea* and *Phytophthora capsici*, respectively. These proteins were found to elicit necrosis, induce resistance, and cause increased production of ethylene as well as the phytoalexin capsidiol in tobacco plants (Ricci et al., 1989; Milat et al., 1991). Elicitin responses were observed in all tested *Nicotiana* spp., but not in other solanaceous species, such as tomato and eggplant. Furthermore, some Brassicaceae species also respond to elicitin; most radish cultivars (*Raphanus sativus*) and one turnip cultivar (*Brassica campestris*), but not *Arabidopsis*, showed necrosis after elicitin treatment (Kamoun et al., 1993). The gene encoding for *P. infestans* elicitin INF1 was found to be downregulated during early infection of potato. However, in the necrotrophic phase of infection *inf1* expression was upregulated (Kamoun et al., 1997). Interestingly, *N. benthamiana*, a nonhost of *P. infestans*, gained susceptibility after silencing of *inf1*, demonstrating that the recognition of INF1 contributes to resistance (Kamoun et al., 1998).

Members of the Peronosporales, e.g., *Phytophthora* spp. and downy mildews are unable to synthesize sterols and must, therefore, acquire them during pathogenesis. Elicitin and elicitin-like sequences are also found in downy mildew pathogens, but no functional analysis has been performed on these proteins (Baxter et al., 2010; Cabral et al., 2011; Stassen et al., 2012; Sharma et al., 2015). Dehydroergosterol binding activity was shown for several elicitins *in vitro*. Furthermore, elicitins are able to catalyze sterol transfer between liposomes (Mikes et al., 1998). However, *in vivo* sterol-binding activity of elicitins has not been demonstrated. Interestingly, the oomycete pathogen *A. euteiches* is able to synthesize sterols and seems to lack elicitin genes (Gaulin et al., 2008, 2010).

The putative elicitin receptor was recently cloned from a wild potato (*Solanum*) that responds to the *P. infestans* elicitin INF1. A *S. microdontum* ecotype showed a clear cell-death response when *inf1* was transiently expressed. Crosses with an unresponsive *S. microdontum* subspecies and further screening and genetic mapping resulted in the identification of the RLP ELR (elicitin response). Stable expression of ELR in *S. tuberosum* cv. Désirée conferred the cell death response after expression of *inf1*. Furthermore, ELR mediated a broad-spectrum response to elicitins of oomycetes: most tested elicitins induced a cell-death response in transgenic ELR potato, even though there is often low sequence similarity between elicitins (Du et al., 2015). Recognition might therefore be based on structural similarity rather than a small conserved peptide. ELR was shown to bind to SERK3/BAK1, but binding of the putative receptor to the RLP adaptor protein SOBIR1 or the elicitin ligand was not tested (Du et al., 2015). Intracellular perception, however, cannot be ruled out as elicitins have, anecdotally, been reported to be detected inside plant cells, e.g., the immunocytochemical localization of the elicitin quercinin in oak (*Quercus robur*) root cells infected with *P. quercina* (Brummer et al., 2002). ELR is thought to mediate extracellular recognition of elicitins, but direct binding to confirm the receptor function of ELR still needs to be demonstrated (Du et al., 2015). Previously, studies in tobacco suggested that INF1 binds to the cytoplasmic domain of a lectin RLK from *N. benthamiana*, NbLRK1 (Kanzaki et al., 2008). Silencing of *NbLRK1* resulted in reduced INF1 responsiveness suggesting the RLK contributes to defense signaling. Although no ELR has been identified in tobacco yet, SERK3/BAK1 and SOBIR1 were found to be required for elicitin-triggered cell death in *N. benthamiana* (Chaparro-Garcia et al., 2011; Peng et al., 2015). It is, therefore, likely that ELR acts similar to RLP23 (Albert et al., 2015) and tomato Cf-4 (Postma et al., 2016), in that it requires both a BAK1-like RLK and SOBIR1-like RLK for pattern-triggered immunity.

#### Cellulose-Binding Elicitor Lectin

A 34 kDa glycoprotein was isolated from *P. parasitica* var. *nicotianae* mycelium that triggered enhanced lipoxygenase activity as well as accumulation the defense-related cell wall hydroxyproline-rich glycoproteins in tobacco. This protein was localized to the internal and external layers of the hyphal cell wall (Séjalon-Delmas et al., 1997). The protein sequence revealed two cellulose-binding domains belonging to the carbohydrate binding module 1 (CBM1) family similar to that of fungal glycanases (Mateos et al., 1997; Gaulin et al., 2006). This putative function was corroborated by demonstrating protein binding to fibrous cellulose and plant cell walls. Furthermore, the protein was shown to have lectin-like activities; human red blood cells were readily agglutinated by this protein. Therefore, it was designated cellulose-binding elicitor lectin (CBEL). Moreover, CBEL was able to elicit necrosis, activate defense gene expression, and trigger immunity to *P. parasitica* var. *nicotianae*. No enzymatic activities for CBEL were observed, suggesting it acts as a pattern (Mateos et al., 1997).

Silencing of *CBEL* resulted in a severe reduction of adhesive abilities of *P. parasitica* var. *nicotianae* to cellulosic surfaces, but did not affect pathogenicity. Interestingly, knockdown mutants showed dispersed abnormal cell wall thickenings, indicating that CBEL might be involved in cell wall deposition in the pathogen (Gaulin et al., 2002). CBEL activity as a pattern is not limited to tobacco, as infiltration of CBEL in *Arabidopsis* leaves resulted in defense responses differentially dependent on the phytohormones SA, JA, and ethylene (Khatib et al., 2004). CBEL-induced necrosis was lost in JA-insensitive *coi1* and ethylene-insensitive *ein2* mutant plants, whereas *PR1* and *WAK1* expression, accumulation hydroxyproline-rich glycoproteins, and peroxidase activity was greatly reduced or abolished in an *Arabidopsis NahG* mutant that metabolizes SA (Khatib et al., 2004). Transient expression of *CBEL* as well as infiltration of recombinant CBEL in tobacco leaves resulted in rapid development of necrotic lesions. Immunocytochemistry revealed that the delivered CBEL was bound to the plant cell wall. Substitution of aromatic residues in CBEL that are possibly involved in cellulose binding reduced the necrosis-inducing activity. Necrosis-induction in tobacco was lost for three recombinant CBEL proteins (Y52A, Y188A, and Y52A_Y188A), that were also unable to induce defense-related genes at similar concentrations as native CBEL. Recently, it was shown that CBM1-1 is the main determinant in the interaction with cellulose; a mutation in CBM1-2 (Y188A) only showed a slight decrease in cellulose binding compared to wild type CBEL, whereas a mutation in CBM1-1 (Y52A) strongly decreased the binding capacity of CBEL and the double mutant (Y52A_Y188A) entirely lost the ability to bind cellulose (Martinez et al., 2015). Taken together, these data show amino acids in the two CBM1s, that were predicted to be important for cellulose binding, are important for elicitor activity.

To define the minimum CBEL pattern that triggers immunity, synthetic peptides of CBM1-1 and CBM1-2 were generated. CBM1-1synt and CBM1-2synt were sufficient to activate plant defense in tobacco and *Arabidopsis*, respectively. Intriguingly, recombinant CBEL but not recombinant CBEL_Y52A_Y188A, induced calcium fluxes in tobacco cells but not in protoplasts. This demonstrates that the plant cell wall and unmodified CBM1s are important for CBEL perception (Gaulin et al., 2006).

CBM1s are probably not essential for pathogens with an obligate biotrophic lifestyle; only one was detected in the *Albugo laibachii* genome and no clear CBM1-encoding genes were found in *H. arabidopsidis*, whereas *Pythium ultimum* and *Phytophthora* spp. contain multiple CBM1-encoding genes (Larroque et al., 2012). It has been proposed that adhesion of CBEL or its CBM1s perturb the cellulose status, and the perception of this disturbance leads to defense activation, but this remains to be proven (Dumas et al., 2008). The fact that BAK1 and RESPIRATORY BURST OXIDASE HOMOLOGUE (RBOH) D and F proteins are required for some of the CBEL-induced defense responses suggests that a PRR might be involved (Larroque et al., 2013). The oxidative burst triggered by pattern recognition is mediated by the NADPH oxidases RBOH D and F (Suzuki et al., 2011). Necrosis-induction by CBEL in *bak1-4* and the *rbohD/F* double mutant was similar to the Col-0 *Arabidopsis* wildtype. However, no ROS production was detected in *bak1-4* and *rbohD/F* and activation of MAP kinases was reduced in *bak1-4* and delayed in *rbohD/F* compared to Col-0. The expression of JA-responsive genes *WRKY11* and *PDF1.2*, but not the expression of the SA-responsive gene *PR1*, was also reduced in these mutant lines (Larroque et al., 2013). The dependence of some CBEL-induced responses on BAK1 suggests a role for an RLK or RLP in the perception of CBEL. Three *Arabidopsis* accessions were found that are unresponsive to CBEL, and may therefore offer a way to decipher CBEL-triggered immunity (Larroque et al., 2013).

#### OPEL

A secreted apoplastic protein from *P. parasitica* called OPEL was recently discovered to trigger a plant immune response (Chang et al., 2015). OPEL contains a thaumatin-like domain, a glycine-rich domain, and a glycosyl hydrolase (GH) domain that has a putative laminarinase active site. OPEL seems to be oomycete specific; homologues were only found in *Phytophthora* spp. and other oomycetes such as *H. arabidopsidis, Py. ultimum* and *A. laibachii*. OPEL is expressed during early infection stages of *P. parasitica*, rapidly increasing transcript levels within 12 hours after inoculation on *N. benthamiana*. Furthermore, infiltration of *N. tabacum* with recombinant OPEL protein resulted in cell death, increased callose deposition, ROS accumulation, induction of defense-related genes and systemic acquired resistance against several pathogens. Moreover, transient expression of *OPEL* in *N. benthamiana* enhanced resistance to *P. parasitica*. It was shown that the GH domain was essential for the increased callose deposition and increased accumulation of ROS in *N. tabacum*. Although the OPEL GH domain contains a laminarinase signature active site motif, no laminarin or β-1,3-glucan enzymatic activity was detected in OPEL recombinant protein. Mutation of the putative laminarinase active site motif in the predicted GH domain abolished elicitor activity of OPEL, which suggests enzymatic activity of OPEL is required for triggering the defense response (Chang et al., 2015). The OPEL substrate has not been identified but is likely a polysaccharide in the plant cell wall. OPEL-released degradation products might, therefore, be perceived by plants as DAMPs.

## Endogenous Patterns

Next to exogenous patterns, host-derived molecules that are released upon pathogen infection can serve as danger signals (**Table 2**). Several endogenous patterns, also known as DAMPs, have been described that are plant cell wall derived or that are released from the host cytosol (Boller and Felix, 2009; Yamaguchi and Huffaker, 2011). The release of these patterns is promoted by a plethora of hydrolytic enzymes that are produced by pathogens (Baxter et al., 2010; Blackman et al., 2015). Interestingly, the downy mildew *H. arabidopsidis* has fewer hydrolases than the hemibiotrophic *Phytophthora* spp., probably as adaptation to its obligate biotrophic lifestyle (Baxter et al., 2010).

**Table 2:** Plant-derived patterns that trigger plant immunity.

Elicitor^a^	Type	Receptor^b^	Receptor type^c^	Source	Reference
Oligogalacturonides	Carbohydrate	WAK1	EGF-like	Cell wall	Ferrari et al., 2013
Cutin monomers	Fatty alcohol	Unknown		Cell wall	Fauth et al., 1998
Peps	Peptide	PEPR1/PEPR2	RLK	Cytosol	Bartels and Boller, 2015
Extracellular ATP	Nucleoside triphosphate	DORN1/LecRK-I.9	LecRK	Cytosol	Choi et al., 2014

*^a^ATP = Adenosine triphosphate.*

*^b^WAK1 = CELL WALL-ASSOCIATED KINASE 1; PEPR1/PEPR2 = PEP1 RECEPTOR 1/PEP1 RECEPTOR 2; DORN1 = Does Not Respond to Nucleotides 1; LecRK-I.9 = lectin receptor kinase clade 1.9.*

*^c^EGF = epidermal growth factor; RLK = receptor-like kinase; LecRK = lectin receptor kinase.*

Oligogalacturonides (OGs) are released from the plant cell wall after mechanical damage or by pathogen-secreted hydrolytic enzymes through degradation of homogalacturonan (Ferrari et al., 2013). OGs bind to several members of the cell wall-associated kinase (WAK) family, which consequently leads to the activation of immunity (Brutus et al., 2010; Ferrari et al., 2013). Also cutin, the main constituent of the plant cuticle (Heredia, 2003), can be degraded to cutin monomers by pathogen released cutinases. Cutin monomers are potent elicitors of defense in several plant species (Schweizer et al., 1996; Fauth et al., 1998). However, it remains unknown how cutin monomers are recognized by plants.

Damage patterns could also be released from the plant cytosol during oomycete infection. These include members of the plant elicitor peptide (Pep) family. The cytosolic precursors of Peps, PROPEPS are released and cleaved when the plant cell is damaged, resulting in the production of endogenous patterns. The receptors for Peps have been identified, the RLKs PEP1 RECEPTOR 1 (PEPR1) and PEP1 RECEPTOR 2 (PEPR2) recognized Peps and contributed to immune responses against several pathogens (Yamaguchi et al., 2006, 2010; Krol et al., 2010; Yamaguchi and Huffaker, 2011; Albert, 2013; Bartels et al., 2013; Bartels and Boller, 2015).

Furthermore, extracellular adenosine triphosphate (eATP) could be perceived as a damage pattern. Treatment of *Arabidopsis* with ATP induced a similar set of genes as wounding did (Choi et al., 2014). In a screen for ATP-insensitivity, a *dorn1* (Does Not Respond to Nucleotides 1) mutant was identified that is defective in the lectin receptor kinase LecRK-I.9. LecRK-I.9 binds to ATP with high affinity and is required for the activation of several ATP-induced responses, demonstrating it is an ATP receptor (Choi et al., 2014). Previously, *lecrk-I.9* mutants were shown to be more susceptible to two *Phytophthora* species than wildtype *Arabidopsis*. Conversely, overexpression of *LecRK-1.9* led to increased resistance to *P. brassicae* (Bouwmeester et al., 2011).

Finally, it has been proposed that recognition of the exogenous pattern β-1,3-glucan could have evolved as an endogenous danger signal; callose could be degraded by host or pathogen-derived β-1,3-glucanases, thereby eliciting a defense response (Klarzynski et al., 2000).

## Putative Receptor Proteins

Plant genomes encode many RLKs and RLPs. The *Arabidopsis* genome, for example, encodes more than 600 RLKs and 57 RLPs (Shiu et al., 2004; Wang et al., 2008). For most of these proteins the function is unknown. We expect that several of these receptor proteins have a role in the perception of oomycete pathogens. Recently, it was shown that many *RLP* genes are upregulated after treatment with *P. infestans* and the *P. infestans* NLP NPP1, suggesting a role for these RLPs during oomycete infection (Wu et al., 2016). Several RLKs are also reported to affect the interaction with oomycete pathogens. For example, other LecRKs, next to the aforementioned LecRK-I.9 and NbLRK1, influence the defense response against *Phytophthora* in *Arabidopsis*, tomato and *N. benthamiana* (Wang et al., 2014, 2015b,a). Silencing of several LecRKs in tomato and *N. benthamiana* led to increased susceptibility to *P. capsici* and *P. infestans*, respectively (Wang et al., 2015b). Two *Arabidopsis* LecRKs from the same clade (IX) were shown to affect *Phytophthora* resistance in a similar way (Wang et al., 2015a). Finally, the *Arabidopsis* LecRK-VI.2A positively regulates the MAMP-triggered immunity response (Singh et al., 2012). Although, some RLKs and RLPs partly regulate the defense response against oomycetes, the patterns or molecules that are recognized by these proteins are still largely unknown.

## Conclusions and Perspectives

Recent discoveries in extracellular recognition of oomycete patterns have provided new insight in how plants detect early infection of these (hemi-)biotrophic pathogens. Novel PRRs for elicitins and NLPs have been identified and mechanisms of how these exogenous patterns are perceived by plants have been elucidated. The scientific progress described in this review provides interesting leads for resistance breeding of crops. For example, transgenic expression of the PRRs ELR and RLP23 in cultivated potato resulted in increased resistance to the late blight pathogen *P. infestans* that is known to produce elicitins and NLPs (Haas et al., 2009; Albert et al., 2015; Du et al., 2015). Classical resistance breeding has mainly focused on the introgression of resistance genes encoding cytoplasmic NB-LRR receptors, which are rapidly broken by new emerging strains of the pathogen. The use of PRRs, many of which recognize conserved microbial patterns, for breeding a new generation of disease resistant crops could offer a more durable solution, especially if PRRs and resistance genes are stacked (Dangl et al., 2013; Schwessinger et al., 2015). A great example is the expression of the *Arabidopsis* PRR EFR in tomato that resulted in broad spectrum resistance to different bacterial pathogens that all produce the EF-Tu pattern that is recognized by EFR (Lacombe et al., 2010). As many of the described oomycete patterns are broadly distributed, expression of the cognate PRRs in crops could reduce plant disease and aid in securing our future food.

## Author Contributions

All authors listed, have made substantial, direct and intellectual contribution to the work, and approved it for publication.

## Conflict of Interest Statement

The authors declare that the research was conducted in the absence of any commercial or financial relationships that could be construed as a potential conflict of interest.
